# Risk factor-based subphenotyping of heart failure in the community

**DOI:** 10.1371/journal.pone.0222886

**Published:** 2019-10-15

**Authors:** Charlotte Andersson, Asya Lyass, Vanessa Xanthakis, Martin G. Larson, Gary F. Mitchell, Susan Cheng, Ramachandran S. Vasan

**Affiliations:** 1 The Framingham Heart Study, Framingham, Massachusetts, United States of America; 2 Department of Cardiology, Herlev and Gentofte Hospital, Hellerup, Denmark; 3 Section of Cardiovascular Medicine, Boston University School of Medicine, Boston, Massachusetts, United States of America; 4 Department of Biostatistics, Boston University School of Public Health, Boston, Massachusetts, United States of America; 5 Sections of Preventive Medicine and Epidemiology, Boston University School of Medicine, Boston, Massachusetts, United States of America; 6 Cardiovascular Engineering, Inc., Norwood, Massachusetts, United States of America; 7 Smidt Heart Institute, Cedars-Sinai Medical Center, Los Angeles, California, United States of America; 8 Department of Epidemiology, Boston University School of Public Health, Boston, Massachusetts, United States of America; Ospedale del Cuore G Pasquinucci Fondazione Toscana Gabriele Monasterio di Massa, ITALY

## Abstract

**Background:**

Heart failure (HF) is a heterogeneous clinical syndrome with varying prognosis. Subphenotyping of HF is a research priority to advance our understanding of the syndrome. We formulated a subphenotyping schema and compared long-term mortality risk among the HF subphenotypes in the community-based Framingham Study.

**Methods and results:**

In hierarchical order, we grouped participants with new-onset HF (stratified by HF with reduced [HFrEF] vs. preserved ejection fraction [HFpEF]) according to the presence of: (1) coronary heart disease (CHD), (2) metabolic syndrome (MetS), (3) hypertension, and (4) ‘other’ causes. Age at HF onset was lowest in people with the MetS (mean 76 vs. 77 years for HFrEF and HFpEF, respectively) and highest in those with hypertension only (mean 82 and 85 years for HFrEF and HFpEF, respectively). For HFrEF, 10-year cumulative mortality and hazards ratios [HR] were 87% for CHD (n = 219; referent group), 88% for MetS (n = 105; HR 0.95 [95% CI 0.73–1.23]), 82% for hypertension (n = 104; HR 0.71 [0.55–0.91]), and 78% for other (n = 37; HR 0.81 [0.55–1.19]). Corresponding 10-year cumulative mortality and HR data for HFpEF were: 85% for CHD (n = 84; referent), 83% for MetS (n = 118; HR 0.98 [0.72–1.33]), 81% for hypertension (n = 127; HR 0.71 [0.52–0.95]), and 76% for other (n = 43; HR 0.76 [0.50–1.14]). In a sample without overt heart failure (n = 5536), several echocardiographic and vascular indices showed graded worsening of age- and sex adjusted-values among those having CHD, MetS, hypertension, or obesity, compared with individuals not having these risk factors.

**Conclusions:**

HF subphenotypes characterized by the presence of CHD or metabolic syndrome present at a younger age and are marked by greater mortality risk. The clinical utility of the proposed subphenotyping schema warrants further research.

## Introduction

Heart failure (HF) is a heterogeneous syndrome in terms of its age at onset, underlying etiology, course of disease and overall prognosis. Recognizing the importance of long-term cardiovascular risk factors for HF risk, the American Heart Association (AHA) has defined 4 HF stages, where stages A and B represent the presence of risk factors and/or subclinical cardiac dysfunction in the absence of symptoms, respectively, and stages C and D represent symptomatic HF of varying severity.[1] Beyond groupings based on preserved versus reduced left ventricular ejection fraction (LVEF), there is currently no consensus on further HF subclassification; such a subphenotyping schema is warranted for better management of HF patients.

**Fig 1:**
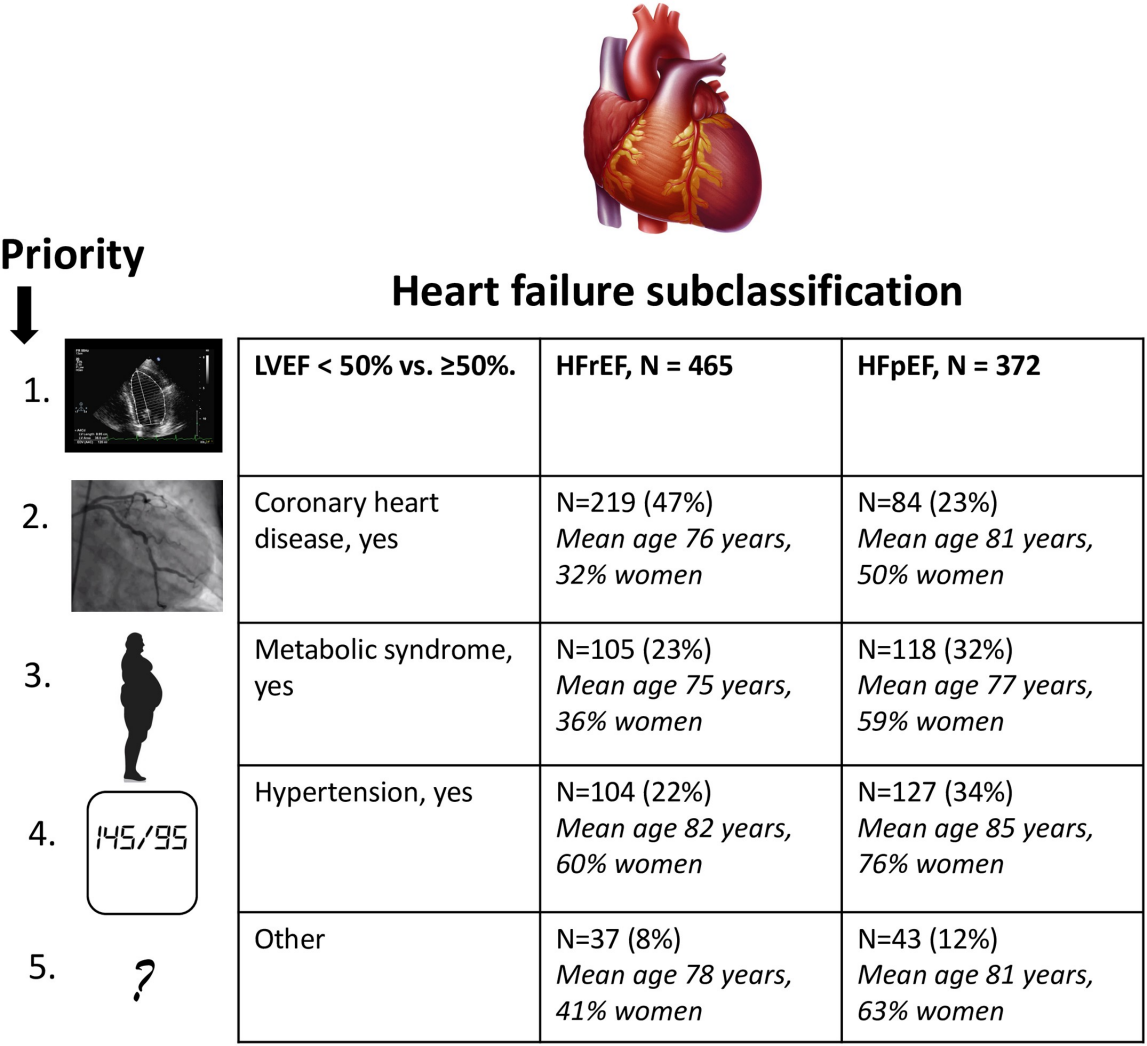
Schematic overview of the HF classification scheme. Table presents age and sex distribution of individuals with HF belonging to the various subphenotyping bins.

Over the last five years, several efforts have been undertaken to subphenotype HF to improve risk stratification and facilitate better management. Many of these strategies have been data-driven (phenomic) or biomarker-based approaches, which have yielded important insights into the heterogeneity of the HF syndrome.[2–5] Some key similarities have been noted across several of the prior studies, including the delineation of prognostically-distinct clusters defined by a high prevalence of the metabolic syndrome, coronary heart disease (CHD), or other risk factor profiles.[2–4] In the present investigation, we extended prior observations on HF subphenotyping by creating a relatively simpler classification system based on a hierarchical etiological schema, as outlined in Fig 1. The central hypothesis underlying the proposed schema is that the presence of a greater burden of cardiovascular risk factors in the community would be associated with an earlier age at onset of HF and subsequently worse clinical outcomes, compared to individuals with a lesser burden of risk factors. As a proof-of-concept investigation to evaluate the construct validity of our suggested approach, we applied the etiological groupings cross-sectionally to echocardiographic and arterial stiffness measures in people without overt HF (corresponding to AHA stage A and B HF), and assessed the mortality-risk associated with the categories prospectively in individuals with new-onset HF (stratified by HFrEF vs. HFpEF) in a community-based sample.

## Methods

### Study samples

The design and selection criteria of the Framingham Heart Study (FHS) cohorts have been described previously.[6–9] In brief, the FHS was founded in 1948 with the enrolment of 5,209 participants from the town of Framingham, MA, USA (corresponding to approximately 20% of the town’s inhabitants at that time) into the Original cohort. Enrolled individuals were between the ages of 30 and 60 years and free of overt cardiovascular disease upon entry into the cohort study. In 1971, children of the Original cohort and the children’s spouses were enrolled into the Offspring cohort (n = 5,124 individuals), and in 2002 the FHS was expanded further with the enrolment of 4,095 children of the Offspring cohort into the Third generation cohort. The age of the participants at study entry has been approximately the same for all three cohorts (young adults). Since enrolment into the FHS, all participants have been examined regularly (approximately every 2–6 years) in predefined examination cycles. For the present investigation, we defined two samples of participants from the Framingham Study Original, Offspring and Third Generation cohorts:

**Sample 1** comprised participants without overt HF at Offspring examination cycle 8 (2005–2008) and Third generation examination cycle 1 (2002–2005).
**Sample 2** included participants with new-onset HF at any of the examination cycles 15 to 28 (1977–2005) for the Original cohort and examination cycles 2 to 9 (1979–2014) for the Offspring cohort. HF ascertainment was adjudicated by panel of three FHS physicians (of whom at least 2 were cardiologists) and a definitive diagnosis of HF required the presence of at least 2 major or 1 major plus 2 minor criteria (Table 1).

**Table 1:** FHS criteria for a heart failure diagnosis.

Major	Minor
Hepato-jugular reflux	Ankle edema
Neck-vein distension (non-supine position)	Night cough
Increased venous pressure (>16 cm H2O from right atrium)	Tachycardia (heart rate >120 beats per minute)
Paroxysmal nocturnal dyspnea	Pleural effusions
Rales in the presence of unexplained dyspnea	Hepatomegaly
Acute pulmonary edema in hospital records	Dyspnea on exertion
A third heart sound (S3, ventricular gallop)	Decreased vital capacity by one third from maximum records
Increased circulation time (>24 seconds from arm to tongue)	
Cardiomegaly and pulmonary hilar congestion at X-ray, or increasing heart size	
Autopsy with evidence of pulmonary edema, cardiomegaly	

A diagnosis of heart failure requires two major, or one major plus two minor criteria.

### Definition of risk factors and comorbidities

Risk factors have been measured routinely on all FHS participants and were defined as being present at any time up to but prior to the onset of overt HF (sample 2), or up to the date of vascular and echocardiographic investigations (sample 1).

Clinical coronary heart disease (CHD) was adjudicated by an endpoints adjudication committee of three physicians who conducted a comprehensive review of all clinical data related to medical history, electrocardiographic, and biomarker findings suggestive of myocardial infarction, coronary insufficiency, or angina pectoris. The metabolic syndrome was defined according to the Third Panel of the National Education Program’s Adult Treatment Panel by the presence of at least 3 of the following: (1) abdominal waist circumference >40 inches in men or > 35 inches in women, (2) blood high-density lipoprotein cholesterol concentration <40 mg/dL in men or <50 mg/dL in women, (3) circulating triglyceride concentration ≥150 mg/dL, (4) blood pressure levels (the average of two separate measures in the FHS clinic) ≥130/85 mm Hg or the use of antihypertensive medications, (5) fasting serum glucose >100 mg/dL or the use of hypoglycemic medications. Hypertension was defined as an examination blood pressure of ≥140/90 mm Hg or the use of antihypertensive medications.[10] We defined chronic kidney disease as an estimated glomerular filtration rate (eGFR) <60 ml/min/1.73 m^2^ or a urine albumin-to-creatinine ratio greater than 30 micrograms/mg.[11] Microalbuminuria was defined as a urine albumin-to-creatinine ratio of 30–300 micrograms/mg.[12] Asthma / chronic obstructive pulmonary disease (COPD) was ascertained based on participant self-report during the FHS examination visits considering all their examinations up to the echocardiography investigation (sample 1) or antedating the HF event (sample 2).

### Echocardiography and tonometry

The echocardiographic measures have been detailed previously and were undertaken in accordance with the American Society of Echocardiography guidelines. In brief, we assessed left ventricular systolic function with LV ejection fraction (LVEF) and longitudinal and circumferential strain (based on speckle tracking imaging). For the longitudinal strain, we used the mean values of the apical 2 and 4 chamber views.[13–15] The circumferential strain was measured in the mid-ventricular parasternal short-axis view.[13–15] All strain analyses were performed by the research team of FHS (comprising 3 trained sonographers; their strain measures have shown to have excellent reproducibility with low inter- and intra-observer variation) using an offline analysis program (2D Cardiac Performance Analysis v1.1, TomTec Imaging Systems, Unterschleißheim, Germany).[14] Frame rates for all analyses were ≥30 frames/sec but <70 frames/sec for all views. We assessed LV diastolic function by E/A ratio, E/e’ ratio (both estimated from the 4 chamber views using pulsed wave and tissue Doppler, respectively), and left atrial end-systolic dimension. LV remodeling was assessed by LV wall thickness, LV end-diastolic dimensions, and LV mass (calculated by the formula of Devereux et al.).[13, 14, 16, 17] Vascular tonometry measures were undertaken by using a customized tonometry system (NIHem, Cardiovascular Engineering Inc), with the carotid-femoral pulse wave velocity (CFPWV) being calculated by dividing the difference in distances from the suprasternal notch to femoral and carotid artery sites by the foot-to-foot transit time of the pulse waveforms obtained from the carotid and femoral arteries.[18]

### Sub-classification schema

For the sample with HF (sample 2), we classified participants as HFpEF vs. HFrEF based on whether their LVEF was ≥50% vs. <50% at the time of or within 6 months after HF onset;[19] both HF types were further divided hierarchically according to the presence of risk factors thus: (1) clinical CHD, (2) the metabolic syndrome, (3) hypertension, and (4) other causes. For the sample without HF (sample 1), a similar classification system was created based on the hierarchy of risk factor prevalence with the proviso that the category “other causes” was replaced by two additional groups, i.e., (4) obesity, and (5) referent group (i.e., those without any of the listed risk-factors).

### Outcomes

Participants with HF (sample 2) were followed for mortality (all-cause and cardiovascular deaths).

#### Ethics approval

All participants gave their written, informed consent at each examination. The study was approved by the Institutional Review Board of the Boston University Medical Center.

### Statistical analysis

Echocardiographic variables that were skewed were natural logarithmically transformed prior to analyses. Additionally, CFPWV was inverse negative transformed prior to analyses (i.e., -1000/CFPWV), and then back-transformed to present the data in original units. The mean levels of echocardiographic and arterial stiffness measures (dependent variables) in the five etiological bins (independent variables) in sample 1 were expressed as least square means derived from multivariable linear regression models that adjusted for age, sex, and cohort type (Offspring vs. Third Generation).

We followed participants from the time of incident HF until December 31, 2015, censoring them at 10 years after their onset of HF. We used age- and sex- adjusted Cox proportional hazards regression models to estimate the cumulative incidence of mortality for each of the etiological subphenotype groups. Cox regression models (adjusted for age, sex, and cohort) were used to compare the mortality risk associated with HFpEF versus HFrEF within each of the etiological subphenotype bins (CHD being used as referent group) after confirming that the assumption of proportionality of hazards was met. We also compared the mortality rates for participants with HF within each subphenotype bin stratified by their HFpEF versus HFrEF status. All analyses were performed in SAS version 9.4 (SAS Institute^®^, Cary, NC, USA). A two-sided p-value <0.05 was considered statistically significant.

## Results

### Association of the scheme with echocardiographic and vascular indices in individuals without overt HF

A total of 5536 individuals without HF with available echocardiographic measures were included in sample 1. The average age ranged from 43 years among the referent group to 68 years for individuals with CHD, Table 2. Those with clinical CHD had lower age-, sex-, and cohort -adjusted least square mean values of LVEF compared with the other subphenotype groups, Table 3. LV wall thickness and LV mass were also higher for the groups with CHD, the metabolic syndrome, hypertension, and obesity when compared with the referent group (without any of these risk factors). The longitudinal strain values were significantly lower in participants within subphenotype bins 1–4 when compared with the etiological bin without risk factors; less pronounced differences between the groups were present for the circumferential strain values (except for those with CHD, who had lower values than the rest). Similar, indices of LV diastolic function (E/e’ ratio and left atrial end-systolic dimension) were worse in all groups compared with the referent group without standard risk factors. The carotid-femoral pulse wave velocity was higher in those with the metabolic syndrome (8.1 cm/s), hypertension (8.0 cm/s) and CHD (7.9 cm/s) compared with the referent group. Similar patterns were evident for the central pulse pressure (Table 2).

**Table 2:** Characteristics of participants without overt HF (sample 1).

	CHD (N = 185)	Metabolic Syndrome (N = 1595)	Hypertension (N = 482)	Obesity (N = 383)	Referent group (N = 2891)
**Age at Index Exam, years**	68.3 (9.6)	56.6 (14.0)	56.8 (14.1)	44.4 (11.8)	43.3 (12.7)
**Women, N (%)**	65 (35%)	732 (46%)	253 (52%)	215 (56%)	1745 (60%)
**BMI, kg/m**^**2**^	28.9 (4.8)	30.4 (5.1)	26.2 (4.4)	33.3 (3.3)	24.1 (2.9)
**Obesity**	69 (37%)	733 (46%)	76 (16%)	383 (100%)	0 (0%)
**Systolic blood pressure, mm Hg**	129 (16)	130 (16)	137 (16)	116 (9)	112 (11)
**Diastolic blood pressure, mm Hg**	70 (9)	78 (11)	82 (11)	75 (7)	72 (8)
**Hypertension treatment, N (%)**	138 (75%)	742 (47%)	272 (56%)	0 (0%)	0 (0%)
**Hypertension, N (%)**	146 (79%)	976 (61%)	482 (100%)	0 (0%)	0 (0%)
**Diabetes, N (%)**	49 (26%)	244 (15%)	0 (0%)	(0%)	(0%)
**Current smoker, N (%)**	16 (9%)	198 (12%)	52 (11%)	39 (10%)	411 (14%)
**Coronary heart disease, N (%)**	185 (100%)	0 (0%)	0 (0%)	0 (0%)	0 (0%)
**Metabolic syndrome, N (%)**	146 (79%)	100%	0 (0%)	0 (0%)	0 (0%)
**Micro albuminuria, N (%)**	28 (15%)	100 (6%)	28 (6%)	11 (3%)	56 (2%)
**Estimated GFR (ml/min/1.73 m**^**2**^**)**	74.4 (17.2)	88.3 (19.0)	87.9 (18.5)	100.5 (15.1)	99.7 (15.9)
**Chronic kidney disease, N (%)**	52 (28%)	203 (13%)	56 (12%)	13 (4%)	86 (3%)
**Atrial fibrillation**	27 (15%)	32 (2%)	6 (1%)	3 (1%)	19 (1%)
COPD*	16 (12%)	65 (8%)	18 (7%)	5 (6%)	38 (7%)

* Percentages are calculated based on the sample where data were available (only in the Offspring sample, at examination cycle 8).

**Table 3:** Association between subphenotypes and various echocardiographic and arterial stiffness measures in individuals without overt HF (sample 1).

	CHD	Metabolic syndrome	Hyper-tension	Obesity only	Referent group	P for difference
N	185	1595	482	383	2891	
Women, N (%)	65 (35%)	732 (46%)	253 (52%)	215 (56%)	1745 (60%)	
LVEF (%)	62.9 (0.006)	65.9 (0.002)	66.3 (0.004)	65.4 (0.004)	65.4 (0.002)	<0.0001
LV wall thickness (cm)	1.90 (0.008)	1.90 (0.003)	1.87 (0.005)	1.87 (0.005)	1.76 (0.002)	<0.0001
LV mass (g)	174 (0.01)	167 (0.005)	160 (0.009)	166 (0.01)	146 (0.004)	<0.0001
LV end-diastolic dimension (cm)	5.1 (0.006)	4.9 (0.002)	4.9 (0.003)	5.0 (0.004)	4.8 (0.001)	<0.0001
MAPSE (cm)	1.57 (0.02)	1.55 (0.006)	1.55 (0.01)	1.63 (0.01)	1.58 (0.004)	<0.0001
E/A ratio	1.22 (0.02)	1.11 (0.06)	1.12 (0.01)	1.16 (0.01)	1.26 (0.005)	<0.0001
E/e’ ratio	6.67 (0.02)	6.39 (0.01)	6.20 (0.01)	6.33 (0.01)	5.70 (0.005)	<0.0001
LA end-systolic dimension (cm)	4.0 (0.008)	3.9 (0.003)	3.7 (0.005)	4.0 (0.006)	3.6 (0.002)	<0.0001
Longitudinal strain (%)	-19.1 (0.2)	-19.5 (0.08)	-19.9 (0.1)	-20.0 (0.1)	-20.9 (0.06)	<0.0001
Circumferential strain (%)	-27.8 (0.4)	-29.9 (0.1)	-29.8 (0.2)	-29.5 (0.2)	-29.6 (0.09)	<0.0001
Carotid-femoral pulse wave velocity (cm/s)	7.9 (0.2)	8.1 (0.05)	8.0 (0.09)	7.4 (0.1)	7.1 (0.04)	<0.0001
Central pulse pressure, pressure flow (mm Hg)	60.2 (0.02)	57.8 (0.006)	62.6 (0.01)	52.6 (0.01)	51.1 (0.005)	<0.0001

Values are cohort, age-, and sex adjusted least square mean with standard errors. CHD, coronary heart disease; LVEF, left ventricular ejection fraction; MAPSE, mitral annular plane systolic excursion.

### Association of the scheme with age at onset of HF and other characteristics

We identified 837 individuals with new-onset HF, of whom 44% had HFpEF. The baseline characteristics for HFrEF and HFpEF are shown in Tables 4 and 5, respectively.

**Table 4:** Characteristics of individuals with HFrEF according to subphenotype category (N = 465).

	CHD (N = 219)	Metabolic Syndrome (N = 105)	Hypertension (N = 104)	Other (N = 37)
**Percentage of all HFrEF**	47%	23%	22%	8%
**Age at CHF, years**	76.1 (10.1)	75.7 (8.8)	82.3 (8.7)	77.8 (10.0)
**Age at Index Exam, years**	76.4 (9.5)	74.2 (8.4)	81.4 (8.5)	76.7 (10.3)
**Women, N (%)**	71 (32%)	38 (36%)	62 (60%)	15 (41%)
**BMI, kg/m**^**2**^	27.1 (4.6)	30.4 (5.4)	24.9 (4.3)	27.1 (5.3)
**Systolic blood pressure, mm Hg**	139 (25)	141 (21)	147 (27)	124 (11)
**Diastolic blood pressure, mm Hg**	70 (13)	71 (13)	69 (14)	69 (10)
**Hypertension treatment, N (%)**	129 (72%)	84 (82%)	82 (79%)	0 (0%)
**Hypertension, N (%)**	151 (83%)	96 (91%)	104 (100%)	0 (0%)
**Diabetes, N (%)**	50 (33%)	62 (61%)	0 (0%)	0 (0%)
**Coronary heart disease, N (%)**	219 (100%)	0 (0%)	0 (0%)	0 (0%)
**Metabolic syndrome, N (%)**	80 (38%)	105 (100%)	0 (0%)	0 (0%)
**Estimated GFR (ml/min/1.73 m**^**2**^**)**	74 (32)	59 (24)	83 (30)	69 (21)
**Atrial fibrillation**	74 (34%)	39 (37%)	32 (31%)	14 (38%)
COPD*	24 (13%)	16 (15%)	11 (11%)	6 (16%)

* Percentages calculated based on the subsample with available data (excluding those with missing values).

**Table 5:** Characteristics of individuals with HFpEF according to subphenotype category (N = 372).

	CHD (N = 84)	Metabolic Syndrome (N = 118)	Hypertension (N = 127)	Other (N = 43)
**Percentage of all HFpEF**	23%	32%	34%	12%
**Age at CHF, years**	81.1 (9.7)	77.3 (10.7)	85.4 (7.1)	80.9 (11.8)
**Age at Index Exam, years**	80.9 (7.5)	76.2 (10.5)	84.4 (6.6)	79.9 (11.2)
**Women, N (%)**	42 (50%)	70 (59.3%)	96 (75.6%)	27 (62.8%)
**BMI, kg/m**^**2**^	27.9 (5.1)	31.6 (6.3)	25.7 (4.3)	26.9 (3.6)
**Systolic blood pressure, mm Hg**	142 (24)	141 (26)	142 (25)	120 (12)
**Diastolic blood pressure, mm Hg**	69 (13)	69 (10)	69 (10)	67 (10)
**Hypertension treatment, N (%)**	52 (80%)	99 (85%)	96 (76%)	0 (0%)
**Hypertension, N (%)**	60 (90%)	109 (93%)	127 (100%)	0 (0%)
**Diabetes, N (%)**	15 (33%)	60 (53%)	0 (0%)	0 (0%)
**Coronary heart disease, N (%)**	84 (100%)	0 (0%)	0 (0%)	0 (0%)
**Metabolic syndrome, N (%)**	27 (33%)	118 (100%)	0 (0%)	0 (0%)
**Estimated GFR (ml/min/1.73 m**^**2**^**)**	71 (29)	64 (24)	73 (22)	92 (32)
**Atrial fibrillation**	39 (46%)	54 (46%)	52 (41%)	26 (60%)
COPD*	6 (9%)	19 (16%)	28 (22%)	8 (19%)

* Percentages calculated based on the subsample with available data (excluding those with missing values).

Individuals with the metabolic syndrome had the lowest age at onset of HF among both the HFrEF and HFpEF strata (mean age 76 vs. 77 years for HFrEF vs. HFpEF, respectively), followed by the groups with CHD (76 vs. 81 years, for HFrEF vs. HFpEF, respectively), while those with hypertension only were oldest (82 vs. 85 years, for HFrEF vs. HFpEF, respectively). The prevalence of other comorbidities (including COPD/asthma and atrial fibrillation) were overall similar across the subphenotype bins for both HFrEF and HFpEF (Tables 4 and 5). For both HFpEF and HFrEF, the proportion of women was highest in the groups with hypertension only, whereas the proportions of men was highest among the groups with CHD.

### Mortality in HF across the various etiological bins

**Fig 2:**
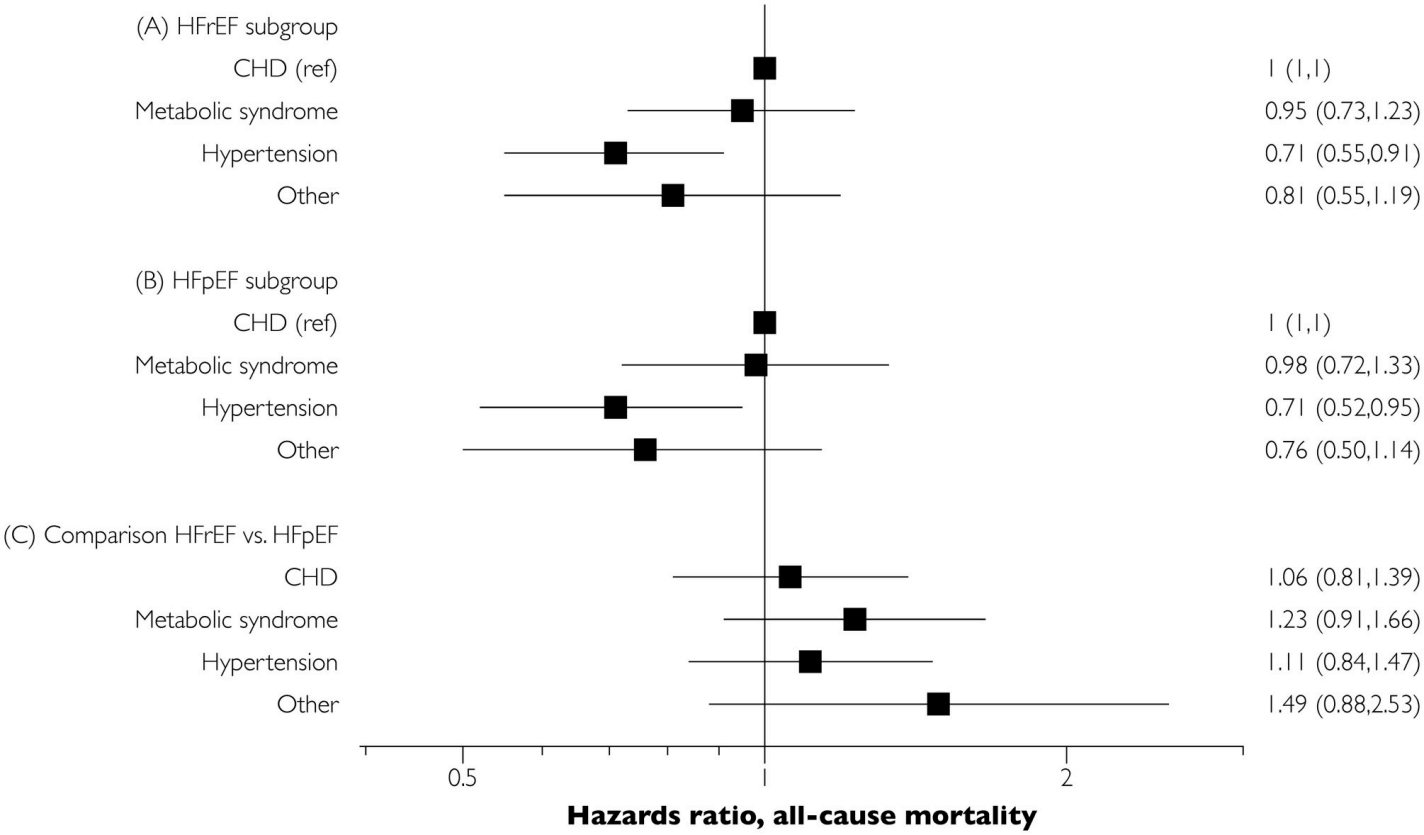
Hazards ratio associated with various subphenotypes for all-cause mortality. Panel A refers to the results from a Cox model including only HFrEF patients, the CHD subgroup served as the referent. Similar, panel B illustrates to the results from a sub-analysis including only HFpEF patients. The panel C shows within each subgroup comparison of mortality risk in HFpEF vs. HFrEF.

Among both the HFrEF and the HFpEF subphenotype groups, age- and sex-adjusted cumulative 10-year all-cause mortality and adjusted hazards ratios were highest for those with CHD and the metabolic syndrome, followed by those with hypertension only and ‘other’ groups (Table 6 and Fig 2). Individuals with hypertension as the only identified etiological factor had a better prognosis relative to those with prevalent CHD or the metabolic syndrome (for both HFpEF and HFrEF subgroups).

**Table 6:** Age and sex adjusted 10-year mortality by group.

Group	HFrEF			HFpEF		
	All-cause Mortality	CVD Death	Proportion of deaths attributable to CVD (%)	All-cause Mortality	CVD Death	Proportion of deaths attributable to CVD (%)
**CHD**	0.87 (0.81, 0.91)	0.75 (0.63, 0.82)	67%	0.85 (0.74, 0.90)	0.62 (0.30, 0.78)	48%
**Metabolic Syndrome**	0.88 (0.78, 0.92)	0.69 (0.50, 0.80)	57%	0.83 (0.75, 0.88)	0.58 (0.36, 0.70)	41%
**Hypertension**	0.82 (0.72, 0.88)	0.68 (0.51, 0.79)	57%	0.81 (0.71, 0.86)	0.47 (0.28, 0.59)	37%
**Other**	0.78 (0.57, 0.87)	0.51 (0.28, 0.66)	59%	0.76 (0.57, 0.85)	0.29 (0.06, 0.48)	30%

Estimates refer to proportions of all individuals who died over 10 years, e.g. 0.87 means 87% were dead after 10 years. CVD, cardiovascular disease.

**Fig 3:**
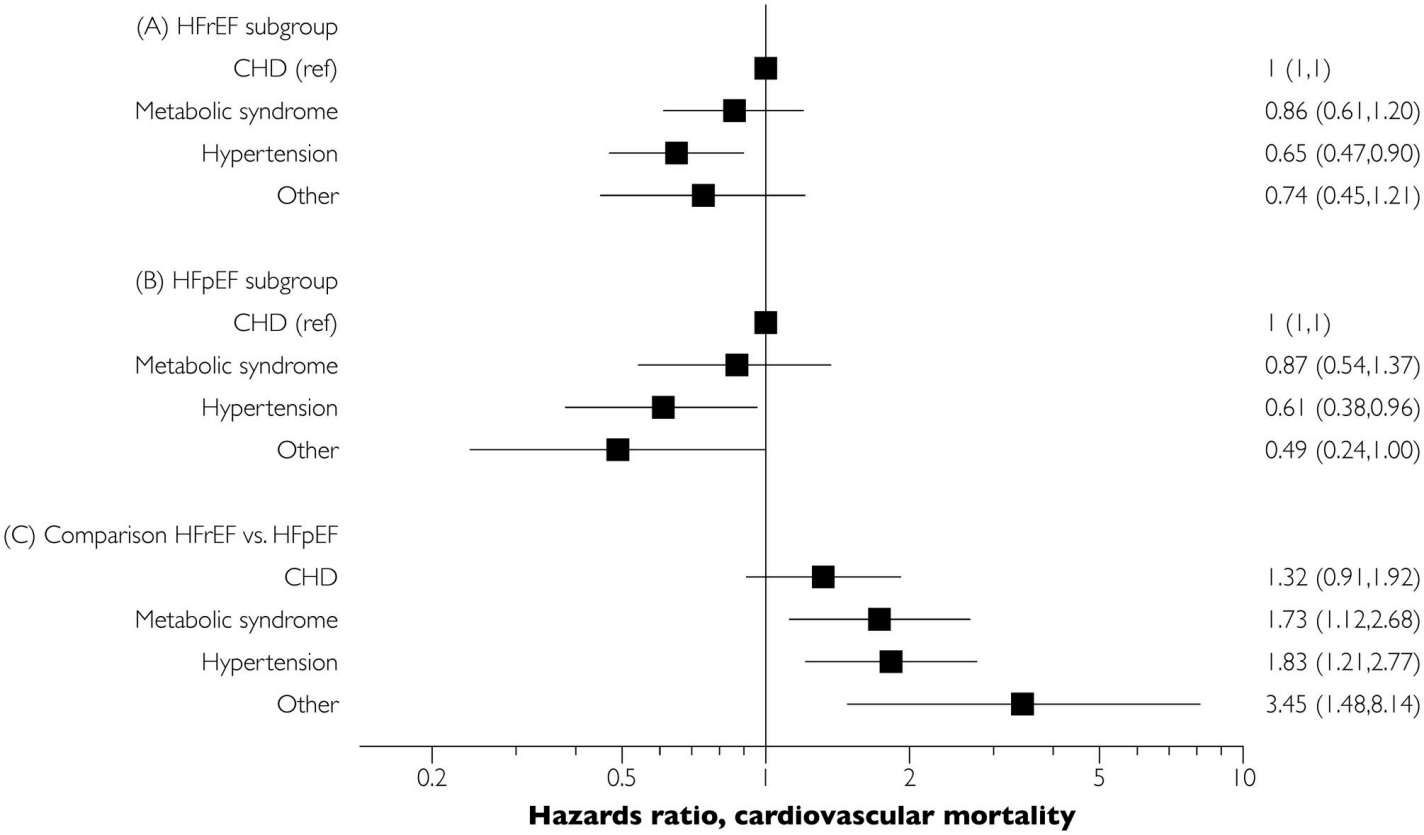
Hazards ratio associated with various subphenotypes for cardiovascular mortality. Panel A refers to the results from a Cox model including only HFrEF patients, the CHD subgroup served as the referent. Similar, panel B illustrates to the results from a sub-analysis including only HFpEF patients. The panel C shows within each subgroup comparison of mortality risk in HFpEF vs. HFrEF.

Regarding cardiovascular mortality, individuals with HFrEF had a higher mortality than individuals with HFpEF for all the subphenotype bins, and a greater proportion of deaths were attributable to cardiovascular disease in those with HFrEF versus HFpEF (Table 6). The observed trend for higher cardiovascular mortality for HFrEF persisted in age- and sex-adjusted Cox regression analyses (Fig 3). With regards to all-cause mortality, the subgroup with hypertension had a significantly lower cardiovascular mortality risk than the CHD subphenotype in both HFrEF and HFpEF categories (Fig 3).

### Sensitivity analyses

We repeated the analyses for the HFrEF subgroup using an LVEF of ≤40% instead of <50%. The results were similar to those of the main models in terms of distribution of individuals in the various risk factor groups, age at HF onset, and mortality risk differences between the subphenotype groups, **online supplemental material**. Further, the group with midrange LVEF (>40-<50%) had comparable characteristics and prevalence of risk factors as the HFpEF and HFrEF groups and an age at onset in between those with HFpEF and HFrEF, although the number of individuals in this group overall was small (n = 102; **online supplemental material**).

## Discussion

In the present investigation, we proposed a hierarchical risk factor-based subphenotyping schema that may be used to complement LVEF for classifying HF in a community-based setting. We demonstrated a greater prevalence of subclinical cardiovascular impairment among individuals without HF who belonged to the first two subphenotyping bins, i.e., presence of CHD or the metabolic syndrome (compared with the other individuals). Among people with clinical HF, the age at onset of HF was lower in the first two subphenotype bins (i.e., the metabolic syndrome and CHD), and their corresponding mortality rates were also higher compared with the other subphenotype groups. Notably, the first three subphenotype bins in our proposed schema captured approximately 90% of all individuals with new-onset HF, suggesting that the classification may hold promise across all AHA’s four HF stages in a community-based setting.

### Construct validity and comparison with previous studies

Our aim was to create a risk factor-based HF subphenotyping schema that would reflect both the risk of developing HF and the subsequent long-term mortality-risk after onset of HF in the community. We hypothesized *a priori* that categorizing people according to their risk factor burden and potency (based on presence of CHD, the metabolic syndrome, and hypertension only vs. presence of none of these risk factors) might be useful for this purpose. In general, the risk of developing HF may be conceptualized as a product of the risk carried by risk factors multiplied by the exposure time. Although we did not directly investigate the association of risk factors with HF risk longitudinally, all HF events were first events. Additionally, participants in the FHS have been followed since young adulthood, justifying the use of age at onset of HF as a proxy for disease propensity associated with the risk factor groupings. Further, among individuals without HF, those belonging to the higher tier of subphenotyping bins (i.e., presence of CHD or metabolic syndrome) had a higher burden of subclinical LV disease measures than those without risk factors, which increases the risk of developing clinical HF. A greater burden of cardiovascular risk factors would not only be expected to translate into earlier HF onset, but also a more rapid progression of disease, as confirmed by greater mortality risk associated with these two groups (compared to the other groups) in our sample. Similar to our study, in the RELAX and TOPCAT trials of patients with HFpEF, those with diabetes and the metabolic syndrome were younger and had a worse prognosis than HF patients who did not have these comorbidities.[20, 21] Further, in prior studies that used more agnostic cluster- and machine learning-based approaches for subphenotyping, the subgroups with a high prevalence of CHD and obesity / diabetes tended to fare worse than some of the other groups without these risk factors.[2–4] Thus, although our classification scheme needs formal external validation, it could represent a first step in the evaluation of potential risk factor burden-based prognostic classifiers of the HF syndrome in the community.

### Differences between HFpEF and HFrEF subgroups and identification of subgroups at high risk of cardiovascular mortality

Within each subphenotyping bin, individuals with HFpEF and HFrEF had similar characteristics and relative mortality risks, although the onset of HFpEF occurred about 2–5 years later than the onset of HFrEF. It is likely that disease risk-modifying factors like sex, genetic variation (e.g., truncating titin-variants), low-grade systemic inflammation, extra-cardiac organ system dysfunction, and history of treatment of cardiovascular risk factors may all have contributed both to the age at onset and the end phenotype (HFpEF versus HFrEF).[22–24] An important difference between HFpEF and HFrEF was that individuals with HFpEF were less likely to die from cardiovascular causes than individuals with HFrEF. Such difference was apparent across all the subphenotyping bins, but was most apparent for the bins with lower risk factor burden (hypertension only or ‘other’ etiology). Slightly less than half of all HFpEF deaths were adjudicated as attributable to cardiovascular causes in the CHD group, as compared with two thirds in the corresponding HFrEF subphenotyping group, although the difference was not statistically significant (Fig 3C). Thus, for both HFpEF and HFrEF, those with a greater burden of risk factors for cardiovascular disease are more likely to die from cardiovascular causes and have a higher overall mortality as compared with individuals without cardiovascular risk factors. These observations are consistent with the existing literature for HFpEF, where highly varying rates have been noted across different reports.[25]

### Perspectives and clinical implications

Although the subclassification into heart failure with preserved versus reduced LVEF (HFpEF versus HFrEF) has been used for identifying drugs and devices that may improve outcomes in HFrEF, this approach has been questioned recently. Furthermore, substantial residual risk is experienced by patients with HFrEF treated according to current guidelines.[26–28] Moreover, clinical trials including a broad spectrum of HFpEF patients have not shown mortality benefit, possibly because of the heterogeneity of the clinical syndrome (including variable burden of the risk factors underlying the HF syndrome) and the relatively larger proportion of individuals dying of non-cardiovascular causes.[24, 29–34] Thus, additional subphenotyping is warranted to better identify HF subgroups at higher risk of cardiovascular mortality. Whether our proposed schema may be useful for this purpose (i.e., to guide the design of future clinical trials) warrants further investigation. The schema may be useful to guide future epidemiological studies, including those evaluating the genetic architecture of HF by yielding clinically homogeneous subsets of HF and reducing phenotypic heterogeneity.[35]

### Strengths and limitations

The present investigation extends prior studies, being based on a well-phenotyped community-based sample with longitudinally collected risk factor data antedating the onset of HF. The longitudinal setting can be important in the assessment of presence versus absence of standard risk factors used for subphenotyping because the prevalence of these risk factors may be confounded by the presence of the HF syndrome or its treatment if such assessment is made at or after HF onset.[36] Yet, it should be noted that our sample was modest in size and comprised of predominantly white individuals of European ancestry. All the participants with diabetes in the present investigation had either CHD or the metabolic syndrome, which precluded a separate group with ‘only diabetes’. Thus, our risk factor-based subclassification system will need external validation to evaluate its utility in other ethnicities. Heart failure was defined according to the Framingham epidemiological research criteria, which may be different from current clinical definitions. For instance, the diagnosis did not involve biomarkers as one of the diagnostic criteria. It is not known if other definitions of heart failure would have altered the number of people with heart failure overall and in different subgroups. Further, although risk factors are likely to contribute significantly to disease onset, not all of them may be directly causal (e.g., CHD may not always be causally related with HFpEF, but act through common risk factors, including hypertension and endothelial dysfunction).[37] Finally, we also did not have advanced echocardiographic examination at the onset of HF in all individuals, or measures of pulmonary vascular function and central arterial stiffness, features that may be helpful to further refine the HF subphenotypes.[38]

## Conclusions and clinical implications

Our proposed risk factor-based subphenotyping using a hierarchical etiological schema for community-based individuals with new-onset HF (or at risk thereof) demonstrated three prognostically-distinct subphenotypes of HF. Among individuals with overt HF, those with CHD or the metabolic syndrome were at the highest risk of death and more often died from cardiovascular causes compared with individuals who do not have these risk factors. Although our classification schema has construct validity, it warrants replication in future studies. If validated, the proposed subphenotyping schema may hold promise for guiding future epidemiological research to improve our understanding of the HF syndrome and potentially aid clinical research by targeting more homogeneous subgroups within this heterogeneous clinical condition.
